# Detecting Micro- and Nanoplastics Released from Food Packaging: Challenges and Analytical Strategies

**DOI:** 10.3390/polym14061238

**Published:** 2022-03-18

**Authors:** Claudia Cella, Rita La Spina, Dora Mehn, Francesco Fumagalli, Giacomo Ceccone, Andrea Valsesia, Douglas Gilliland

**Affiliations:** European Commission, Joint Research Centre (JRC), Ispra, Italy; claudia.cella@ec.europa.eu (C.C.); rita.la-spina@ec.europa.eu (R.L.S.); dora.mehn@ec.europa.eu (D.M.); francesco-sirio.fumagalli@ec.europa.eu (F.F.); giacomo.ceccone@ec.europa.eu (G.C.); andrea.valsesia@ec.europa.eu (A.V.)

**Keywords:** microplastics, nanoplastics, microplastic detection and identification, microplastic quantification, food packaging, particle release, plastic consumption

## Abstract

Micro- and nanoplastic (pMP and pNP, respectively) release is an emerging issue since these particles constitute a ubiquitous and growing pollutant, which not only threatens the environment but may have potential consequences for human health. In particular, there is concern about the release of secondary pMP and pNP from the degradation of plastic consumer products. The phenomenon is well-documented in relation to plastic waste in the environment but, more recently, reports of pMP generated even during the normal use of plastic food contact materials, such as water bottles, tea bags, and containers, have been published. So far, a validated and harmonized strategy to tackle the issue is not available. In this study, we demonstrate that plastic breakdown to pMP and pNP can occur during the normal use of polyethylene (PE) rice cooking bags and ice-cube bags as well as of nylon teabags. A multi-instrumental approach based on Raman microscopy, X-ray photoelectron spectroscopy (XPS), scanning electron microscopy (SEM), and particular attention on the importance of sample preparation were applied to evaluate the chemical nature of the released material and their morphology. In addition, a simple method based on Fourier transform infrared (FT-IR) spectroscopy is proposed for pNP mass quantification, resulting in the release of 1.13 ± 0.07 mg of nylon 6 from each teabag. However, temperature was shown to have a strong impact on the morphology and aggregation status of the released materials, posing to scientists and legislators a challenging question: are they micro- or nanoplastics or something else altogether?

## 1. Introduction

The spread of microplastic pollution across our planet may be likened to the sword of Damocles: a threat which grows while knowledge gaps—due to inadequate analytical tools—continue leave us uncertain of when and how the negative effects may be manifest [1,2,3].

It is now well established that particulate microplastics (pMP) are present in detectable concentrations in the oceans, surface waters, soil, and atmosphere, and researchers suspect that they might harm the ecosystem [4,5,6,7,8]. In contrast, the real-world occurrence of particulate nanoplastics (pNP, i.e., typical size of less than 1 µm), even in simple matrixes, remains largely unknown due to the lack of effective, harmonized, and commonly available analytical methods tailored to the nano-range [9,10,11,12,13,14].

The precautionary principle is promoting a number of actions across the globe to tackle pMP release in the environment [15,16,17]. On the one hand, actions to improve our knowledge on biodegradable plastics and increment their use are promoted from both scientific and policy points of view, especially in the framework of the circular economy and end-of-life management routes [18,19,20]. Additionally, in 2016, the European Food Safety Authority (EFSA) highlighted the need to monitor the occurrence of pMP in food products and the development of validated methods for their detection and quantification [21]. This call has been reiterated in the recent literature [22,23,24,25]. pMP in food are typically thought to originate from contaminated environmental sources, as demonstrated for seafood, such mussels and krill [23,26] and fish [26,27]. Beverages, such as beer, honey, and drinking water were also reported to contain microplastics [24,25,27,28,29,30,31] with various suspected origins. In particular, for bottled water, many of the identified pMP were found to be chemically identical to the food packaging material [28,29,31,32,33,34]. Emerging studies additionally reported the release of plastic particulates from a variety of food contact materials and especially food packaging [35,36,37,38,39,40,41,42].

The use of plastics in food packaging has undoubtable had an enormous positive impact on human life by helping in food preservation, while its low density and weight provide benefits for transport and logistics. The polymers in food packaging materials are generally considered to be chemically inert, and the legal controls of material migration into food products consider mainly low molecular weight compounds (<1 kD), such as unreacted or partially unreacted monomers, processing aids, and additives. For such controls, standardized methods have been developed in order to simulate typical food contact scenarios and components [43,44]. However, most of the methods focusing on the release of additives and oligomers are chromatography- and/or mass spectrometry-based and include a filtration step in the analysis process. This filtration, even unintentionally as in the case of HPLC column guards, serves mainly as instrument protection but essentially prevents the detection of particulate plastic material in food contact experiments [45,46].

From various publications reporting the release of pMP and pNP from food packaging, it appears that there is still an absence of any common or systematic analytical strategies, and authors have explored different approaches for particle detection and characterization. In some cases, chemically specific methods, such as Fourier transform infrared (FT-IR) microscopy and pyrolysis gas chromatography/mass spectrometry, have been used, while others report non-specific methods, such as scanning electron microscopy (SEM), nanoparticle tracking analysis, fluorescent microscopy, or quartz crystal microbalance [35,36,37,38,39,40,41,42]. Recently, a call for more knowledge and experimental data generated under realistic scenarios was conducted, with a particular focus on the assessment of the amount of pMP and pNP [14], and the additional request to improve study comparisons by developing and validating certified reference materials that can mimic pMP and pNP in the environment [14,47].

In this report, we will present the results of an investigation of pMP and pNP release from polyethylene (PE) rice cooking bags, ice cube bags, and nylon teabags, examples of everyday food packaging subjected very different use conditions. Our working scheme for sample treatment, based on a multi-instrumental approach, took advantage of the existing literature about pMP and pNP in the environment [48]. In particular, we will discuss critical preparation steps to minimize particle agglomeration, thus improving the detection of individual particles (nanometer and micron-sized). We especially focus on spectroscopic techniques, such as Raman microscopy (µRaman), as key methods for the identification of plastics and other materials coming from the packaging, such as dyes or food residues. Moreover, in case of higher quantities of polymer release, we demonstrate how classical FT-IR spectroscopy using potassium bromide (KBr) pellets can be a simple and useful tool for plastic particle mass quantification. Finally, the same approach was explored as a fast and cost-effective tool to determine the structural nature of the released material: oligomers vs. pMP/pNP.

## 2. Materials and Methods

### 2.1. Measures to Avoid Contamination

Ultrapure water (MilliQ, Millipore, filtered 0.2 µm) was used in all the experiments. In order to reduce sample contamination coming from the laboratory environment, plastic labware was avoided wherever possible. All glassware and metallic items were cleaned with Hellmanex^®^ III detergent (Sigma Aldrich, St. Louis, MI, USA) 2% *v*/*v*, then rinsed with MilliQ water. Cotton lab coats were used. In order to estimate the contribution of airborne contamination, procedural blanks were performed as described.

### 2.2. Sample Preparation

Ice cube bags. Commercially available ice cube bags were filled up with 300 mL of MilliQ water and placed in a freezer (−20 °C) for 24 h. The ice cubes were then manually released from the bag and transferred into a clean glass beaker. After thawing at room temperature, the water was filtered through an Anodisc filter with a 0.1 µm pore size (25 mm with a polypropylene ring, Sigma Aldrich). As a negative control, ice cube bags were filled with the same amount of water, stored at room temperature for 24 h, and filtered as described above. Filters were placed on a glass support and analyzed by µRaman.

Rice bags. Commercially available cooking bags containing rice were opened with clean metallic scissors. After the removal of rice, the bag was placed in 250 mL of boiling water in a 500 mL glass beaker. Following the instructions for preparation time indicated on the packaging, the bag was kept under the liquid surface at 100 °C for 10 min with a glass rod. The bag was then removed from the beaker and the remaining liquid was filtered through an Anodisc filter with a 0.1 µm pore size. A blank control experiment was performed in parallel by boiling the same amount of MilliQ water for the same time and filtering the liquid on the same type of filter. Filters were placed on a glass support and analyzed by µRaman.

Teabags. Commercially available empty plastic teabags were purchased from an online platform. The attached strings with paper labels were removed before proceeding. Each teabag was steeped at 95 °C for 10 min in 10 mL of MilliQ water. The teabags were removed and squeezed with the help of a metallic tweezer. The obtained leachate was freeze dried (Martin Christ, Model Alpha 1–4 LD plus) after a pre-cooling period of 2 h at −20 °C and a freezing step in liquid nitrogen for some minutes. Residues were resuspended in 1 mL of fresh MilliQ water and immediately used for further analysis. Negative controls were prepared with the same procedure without the heating step. For µRaman mapping, 1 µL of the resuspended leachate was pipetted on a clean silicon wafer and allowed to dry at room temperature. Additionally, µRaman measurements were performed on the solid residues obtained by filtering the teabag leachate through Si_3_N_4_ filters (pore size 0.45 µm, Aquamarijn, The Netherlands). The filtrate that passed through the pores was collected and spotted on a Si-PTFE-PDDA chip using a microspotter (Sci Flexarrayer, Scienion) (drop volume 300–360 pL, 1000 drops spotted). The resulting spots (approx. 0.8 mm in diameter) were viewed by the optical microscope and then characterized by µRaman.

### 2.3. Identification Methods

Raman spectroscopy. A WiTec alpha 300 Raman microscope equipped with a 532 nm laser was used to analyze the original food packaging and the released particles. The spectra of the original materials were collected using either 10× or 100× objectives by averaging at least 10 spectra. Spectral recognition was performed with the help of the OpenSpecy database [49,50] and by comparison with a home-built polymer database (UVIR Manager, ACD labs, Toronto, ON, Canada). Pigment identification (in case of the ice cube bags) was conducted using the open-source database of the Pigments checker of Cultural Heritage Science Open Source [51] and the UVIR manager software. For the rice cooking bag samples, Raman mapping of the rice and polymeric particles on Anodisc filters was performed over a 150 μm × 150 μm area at a 2 μm resolution (step size) using a 10× objective and a 2 s integration time. In the case of ice cube bags, a Raman analysis on the Anodisc filters was carried out over a 31 μm × 31 μm area at a 1 μm resolution (step size) with a 10 s integration time and a 100× objective. The same parameters were used also for the teabag leachate deposited onto a silicon substrate. The WiTec Project software was additionally used to generate 2D chemical maps based on the base component analysis or peak signal intensity.

X-ray photoelectron spectroscopy (XPS). XPS measurements were performed with an Axis Ultra spectrometer (Kratos, Manchester, UK) using a Kα Al monochromatic source (hν = 1486.6 eV) operating at 150 W and an X-ray spot size of 400 μm × 700 μm in the hybrid mode. The residual pressure of the analysis chamber during the analysis was less than 8 × 10^−9^ Torr. For each sample, both survey spectra (0–1150 eV, pass energy 80 eV) and high-resolution spectra (pass energy at 40 eV) were recorded. The surface charge was compensated by a magnetic charge compensation system, and the energy scale was calibrated by setting the C 1s hydrocarbon peak to 285.00 eV in binding energy [52,53]. The data were processed using vision2 software (Kratos Analytical, Manchester, UK), and the analysis of the XPS peaks was carried out using a commercial software package (CasaXPS v2.3.18PR1, Casa Software, Ltd., Teignmouth, UK). Peak fitting was performed with no preliminary smoothing. Symmetric Gaussian-Lorentzian (70% Gaussian and 30% Lorentzian) product functions were used to approximate the line shapes of the fitting components after a Shirley-type background subtraction. In order to produce relatively wide, uniformly covered surfaces suitable for XPS analysis, samples were diluted in MilliQ water, drop-cast, and vacuum-dried in triplicate on clean Teflon substrates.

### 2.4. Quantification Method

Application of Beers law to Fourier Transform Infrared (FT-IR) spectroscopy. In order to quantify the plastic material released from the food contact containers during use, we adopted a general approach using Beer’s law and FT-IR spectral analysis as described in detail in Figure S1.

For this study, specific calibration series were created with nylon 6 (nominal range 5–50 µm, Sigma Aldrich) or PE powder (Mw 4000 Da, Sigma Aldrich) for teabag leachate and rice bag samples, respectively. Due to their micron size, the nylon 6 particles were mixed with KBr powder in a mass ratio of 1:100 and micronized by cryo-milling in a SPEX 6875 Freezer/Mill. Known amounts of the plastic-containing KBr powder were weighed and mixed with additional KBr before being mechanically pressed to create 133.7 ± 3.9 mg pellets containing 0.64 ± 0.01/0.09 ± 0.01 mg of nylon 6 plus a plastic-free KBr pellet for use in background estimation (n = 4, values are presented as means ± standard deviations). In the case of polyethylene, calibration points were registered in the 2.00–0.13 mg PE/pellet range plus a plastic-free KBr pellet. Polyethylene residues were quantified based on the peak at about 720 cm^−1^ (CH_2_, rocking), while for nylon 6, the region between 1490 and 1810 cm^−1^ was selected. In all cases, the FT-IR spectra were collected from 4000 to 600 cm^−1^ with a resolution of 4 cm^−1^ (Tensor27, Bruker, Bremen, Germany).

For the preparation of the pellets of residues from the heat-treated rice bag samples, 5 mL aliquots of the cooking liquid were pipetted into glass test tubes, and 200 mg of KBr was added to each tube. Each sample was then lyophilized and carefully mixed before 130 mg of the resulting powder was used to produce KBr pellets.

For the teabag samples, 400 mg of KBr was added to the leachates, and the salt was allowed to completely dissolve before freeze drying (n = 6). Multiplication with the appropriate dilution factor allowed the total amount of nylon 6 in the original teabag leachate to be determined. A procedural blank was obtained by performing the complete procedure without steeping any teabags.

Additionally, teabag leachates used immediately after preparation (“hot-filtered”) or after cooling at room temperature (“cold-filtered”) were filtered with different pore sizes, namely, Anodisc filter 0.02 µm, Anodisc filter 0.2 µm, silver filter 0.45 µm, and polycarbonate track edge filter 2.0 µm (Figure S2). All the filters were 25 mm in diameter and were purchased from Sigma Aldrich. The liquid that passed through the filter into the receiving flask was mixed with 300 mg of KBr and freeze dried. The resulting powder was used to create a pellet as previously described. All the data are presented as means ± standard deviations.

### 2.5. Statistical Analysis

The effect size was estimated in our study by means of Cohen’s d value. Cohen’s d expresses the size of the difference between two observable variables independent from the sample size. The definition [54,55] used in this work is represented in Equation (1):

(1) $$\mathrm{Cohen}\prime s\;d\;=\;\frac{{\mathrm{mean}}_{1}\;-\;{\mathrm{mean}}_{2}}{\sqrt{\frac{SD_{1}^{2}\;+\;SD_{2}^{2}}{2}}}$$

where mean_1_ and mean_2_ refer to the two variable means, while *SD*_1_ and *SD*_2_ represent their respective standard deviations. In accordance with Sawilowsky [56], *d* > 0.8 was considered as the boundary for a “large” effect size, implicating both (i) meaningfulness of the observed difference between the measured means and (ii) sufficient statistical power of the sample to support a subsequent significance test. To estimate the statistical significance of the difference between the two measurement groups, we performed a two-sample *t*-test (Origin, Version 2019b, OriginLab Corporation, Northampton, MA, USA).

### 2.6. Morpholigical Study

Scanning Electron Microscopy (SEM). SEM images were obtained by a FEI-NovaNanolab 600I microscope by detecting the secondary electrons at a 5 kV acceleration voltage with different apertures. Two different approaches were used to prepare the teabag leachate samples for direct imaging by SEM. In the first method, a drop of the sample was spotted and allowed to air dry on a clean silicon substrate previously rinsed with ethanol. In the second method, 10 µL of phosphate-buffered saline solution (Sigma Aldrich) was added to 90 µL of leachate sample and then spotted onto a silicon surface whose hydrophobicity and charge had been modified (Si-PTFE-PDDA) as described in the literature [57,58]. Briefly, a polytetrafluoroethylene (PTFE) coating was plasma-deposited to generate a hydrophobic surface. The deposition was performed using pure octofluorocyclobutane (C_4_F_8_) as the gas precursor at a pressure of 3.5 Pa (27 mTorr), applying a power of 142 W for 5 min. The plasma-modified substrate was incubated for 2 min in poly(diallyldimethylammonium chloride) (PDDA, Sigma Aldrich, Milan, Italy) 2% *v*/*v* before rinsing with MilliQ water and drying under nitrogen flow. Spotting was conducted by pipetting 1 µL of solution in triplicate. In order to allow the time for adsorption of the particulates from the liquid onto the surface, the samples were incubated in a closed box overnight at 4 °C at a relative humidity >85%. Then, they were gently rinsed with MilliQ water to remove any unbound particles and/or any soluble residues.

## 3. Results and Discussion

The identification and quantification of the micro- and nanoplastics released from food contact materials and food packaging is still considered a challenge despite the increasing number of literature reports on the topic [36,37,38,39,40,41,42].

To assess if and how food packaging can release pMP and pNP when subjected to their normal, everyday use conditions, we focused on a selection of three food packaging types: PE rice cooking bags, ice cube bags, and nylon teabags. These food containers were chosen partially on the basis of their widespread use but also because their normal handlings expose them to conditions well beyond normal ambient temperature. Moreover, we want to concretely answer to the need of protocol harmonization in the field by adopting a multi-instrumental approach as already validated in the environmental field [48].

The first step that was considered in development of our testing strategy was to ensure that the particles released were produced under conditions that mimic their intended use. In general, the scenarios of the everyday use of food packaging include the application of direct heating, microwave irradiation, freeze-thaw cycling, or steeping directly in boiling water. In this study, given the intended use of the selected containers, water was selected as the most appropriate food simulant, as indicated by the Council Directive 85/572/EEC [43]. The food packaging was treated with water under hot conditions (boiling water) in the case of nylon teabags and rice cooking bags or with a freeze-thaw process in the case of PE ice bags. In contrast to some other cases reported in the literature [21,59], this removed the need to eliminate the organic contamination that could hinder correct particle counting and identification.

In all investigated cases, it was necessary to concentrate and collect the released particles on a surface suitable for further analysis. Freeze-drying was found to be a good method for concentrating small volumes of liquid as in the case of teabag leachates. For later analytical investigations, it was possible to redisperse the lyophilized powder in an appropriate medium or to use the powder directly as described later in the text. To our knowledge, few other authors have explored solvent evaporation [48,60] or freeze-drying as methods to concentrate pMP and pNP for analytical studies. The main drawbacks of such concentration steps include the risk of aggregation and a possible loss of particles, either by adhesion to the vessel walls or by flying away due to repulsion by electrostatic charges, which are especially relevant in the case of the few particles present in a large amount of liquid. In our case, the freeze-drying process was further explored as a preparation step for the quantification of released materials, as will be explained later in the text. When the volume was higher than 100 mL, as in the case of PE ice bag and PE rice bag sample preparation, filtration was preferred as the concentration method. Alumina filters with a pore size of 0.1 µm were selected because of their suitability for use with different spectroscopic techniques, e.g., as a support for µRaman while their low absorption of IR light above 1500 cm^−1^ makes them acceptable for use with µFTIR in transmission mode.

**Figure 1:**
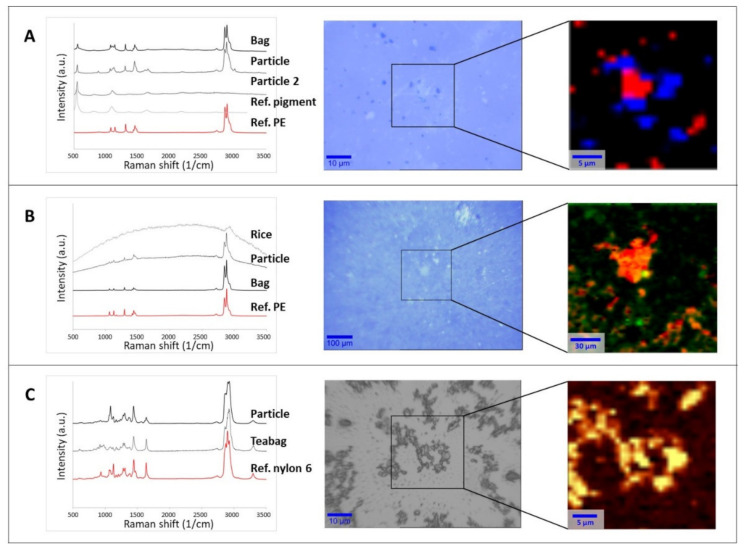
Raman spectral identification (left), optical image (middle), and chemical heat maps (right) of the collected particles released from food packaging materials: (**A**) ice bag, (**B**) rice cooking bag, (**C**) teabag. The rectangular area in the optical images shows the area analyzed by 2D mapping and the subsequent base component analysis or intensity peak heat map. Chemical maps of the filtrate of the ice bag and cooked rice bag on Anodisc filters were generated by base component analysis. For the ice bag (**A**), the red and blue colors identify different particle types (PE and ultramarine blue pigment, respectively). For the rice bag (**B**), the orange and red color scale represents the PE signal intensity, while the green dots indicate rice residues. The chemical heat map of the teabag filtrate on a silicon support (**C**) was generated from the intensity of the signal between 1611 and 1688 cm^−1^ (amide I, C=O stretching). In the color scale here, yellow represents the strongest signal coming from the polymer with respect to dark brown (the lowest signal).

Particles collected on supports (filter surface or silicon substrate) were then analyzed by µRaman to assess their chemical nature and establish if they were actually released from the food packaging (Figure 1). Spectral matches with reference materials in both the open-source [49,50] and home-built libraries were higher than 0.95 in all the tested cases. Additional comparisons were made with the spectra of the original packaging. Figure 1 shows three selected examples with optical images of particles and spectral identification.

Figure 1A (middle panel) refers to the particles released from the water frozen in ice cube bags. The image illustrates the presence of at least two populations of particulates with different colors (white and dark blue in the middle optical image). Raman analysis of the white particles provided spectra where most of the peaks were characteristic of PE, which was the raw material of the plastic bags. For the full assignment of the additional peaks, it was necessary to also consider the analysis of the dark blue particles, attributable to the ultramarine blue pigment generally used to dye food packaging [61,62]. In this case, the characteristic spectral peaks of the pigment at 548, 1096, and 1647 cm^−1^ [63] were also present in the spectra of the white particulates. The analysis of different spots and the chemical mapping of a selected area (Figure 1A, right panel) on the filters confirmed that the released material was composed of PE particles mixed with the pigment (red area in Figure 1A right panel) and individual particles of the ultramarine blue pigment (blue area in Figure 1A right panel). This suggests that both components are likely to be present when the ice cubes are prepared and, consequently, could be ingested. As a control, the same analysis was carried out using water samples which contacted the plastic only at room temperature. In this case, only a few particles were visible on the filter, and they were mainly recognized as PE. The difference of the control and the frozen sample suggests that the release of PE and ultramarine blue pigment was mainly due to mechanical stress, either during the freezing process or when removing of the ice cubes from the bags.

As in the case of freezer bag, the water used to boil the rice cooking bags was filtered on Anodisc filters, leaving a clearly visible layer of particulate on the filter surface (Figure 1B, middle panel). Spectra attributable to PE (the material of the selected rice bag) were detected at different locations on the filter. However, some of the measured spectra showed a strong fluorescence, similar to that observed in the analysis of the rice itself. Fluorescence due to the presence of organic compounds when performing Raman investigation at short wavelength excitation is a well-known phenomenon in the literature [64,65]. Although the rice had been removed from the packaging before cooking, a base component analysis of the spectral map revealed that spectra fully or partially attributable to rice were randomly present (Figure 1B, right panel). This co-localization of spectra suggests that hydrophobic PE particles released from the bag will probably be adsorbed on the rice during typical cooking process. In the blank sample, no particles attributable to rice or PE were found on the filter. The chemical recognition of the particles is a fundamental step before proceeding with any further investigation, as already pointed out in the recent literature [14,23,48]. As presented here, not only pMP and pNP can be present in a sample but also food residues, pigments, and additives associated with the plastic packaging.

In the case of the material released from the teabags after steeping in water, the freeze-dried leachate was resuspended in MilliQ water and spotted on a silicon support. As can be seen in the middle panel of Figure 1C, only one component was detected in the sample, namely, nylon 6 (the material of the original teabags). In some cases, fluorescence was observed, probably due to a weak auto-fluorescence of the original material (data not shown). Figure 1C (right panel) illustrates the chemical heat map created on the basis of the intensity signal of the peak between 1611 and 1688 cm^−1^, which is characteristic of the amide group. Since this chemical group is present only in the polyamide (nylon) and polyurethane families, its presence reasonably indicates that all the detected particles were associated with the nylon released by the original teabag due to the heating process.

Additionally, the chemical nature of the released particles in different particle size ranges was investigated. To conduct this analysis, teabag leachate was filtered through a Si_3_N_4_ filter (0.45 µm pore size) to produce two distinct particle size fractions, namely, below and above the filter cut-off. The particulates larger than 0.45 µm retained on the filter surface (residue) were directly analyzed with µRaman, while the filtrate liquid containing the smaller particles was microspotted on a silicon surface to concentrate the solids into a small uniform spot prior to the Raman analysis. In both cases, the spectra matched that of the native teabag material (Figure S3). A microspotter is an effective tool to deposit small quantities of dispersed solids into very small, well-defined areas for microanalysis. Moreover, by concentrating many particulates into one highly localized and well-defined position, a spectrum can be obtained that is the sum of the signals coming from many particulates. In this way, µRaman characterization becomes feasible, even when the individual particle size is expected to be below the instrument’s resolution and sensitivity [66]. As far we know, there are no descriptions in literature of the use of this tool for pMP and pNP investigation.

The sub-micron particulate fraction (pNP fraction) obtained after filtration was further analyzed by XPS spectroscopy and compared with the surface chemistry of the pristine teabag. The spectra and quantification of the elemental surface composition for both the pNP fraction and the teabag surface are shown in Figures S4 and S5 and Table S1. In both cases, the dominant peaks detected in the spectra were assigned to C1s, O1s, and N1s. This set is compatible with the Raman spectral assignment for nylon. In the pNP fraction spectrum, several minor low-intensity peaks were also observed, and they were assigned to trace contaminants (Na 1s, Ca 1s, Si 2p, B 1s, P 2p, and S 2p), either resulting from the water dispersion, sample preparation, or airborne contamination. In both analyzed samples, the measured stoichiometric elemental ratio N/C is compatible with the theoretical value expected from the nylon composition (Figure S6). The O/C elemental ratio measured on the teabag net is also compatible with nylon assignment, but, in the case of pNP, a much larger value was obtained. It is likely that the large specific surface area of the nano-fraction filtered material was readily oxidized when exposed to air and MilliQ water during sample preparation, a phenomenon already described in the literature [48]. In addition, Nylon hydrolysis could increase the O/C ratio by forming –COOH groups, which would probably be concentrated at the surface of the particles, making them more hydrophilic. The surface oxidation occurring in the pNP fraction was also confirmed by the high-resolution scans performed over the C1s peaks (Figure S7). The slight underestimation of the measured N/C values in the two samples and the slight overestimation of the O/C ratio in the case of the teabag net, as compared to the theoretical values, indicated a small but detectable amount of organic contamination on the material’s surface. Even if XPS alone is not able to unambiguously identify the polymer type or to completely exclude the presence of other components, the overall characterization of the sub-micron fraction of the teabag leachate indicated that it is mostly represented by nylon 6.

In order to quantify the plastic material released from the food packaging during use, we adopted the approach described in the Materials and Methods Section of using Beer’s law and FT-IR spectral analysis.

PE residues were quantified based on the peak at around 720 cm^−1^ (CH_2_ rocking), while for nylon 6, the region between 1490 and 1810 cm^−1^ was selected because it is specific for amide peaks (amide I C=O stretching and amide II C–N stretching and N–H bending) and it is less affected by the overlay with the OH peak in the region around 3200–3300 cm^−1^ (NH stretching). The calibration curve for nylon 6 in the selected mass range (0.65–0.00 mg nylon 6/pellet) showed good linearity and a Pearson correlation coefficient of 0.9982 (Figure S8). From the teabags, a release of 1.13 ± 0.07 mg of nylon 6 (n = 6) was found, as can be seen in Table S2. This corresponds to the release of 5.98 ± 0.81 mg/g of the original teabag. The value in terms of mg of nylon 6 obtained with the procedural blank was less than the 3% of the nylon 6 found in the teabag leachates. This also includes the potential contamination deriving from the condensation of atmospheric humidity during the freeze-drying procedure.

The same approach was also used for the materials released by the rice bags, although, in this case, the amount of PE residue was below the detection limit in the KBr pellets (Figure S9). Similarly, for the particulate released from the ice bags, this approach was not applicable due to the low quantity of material released.

Commonly available methods for pMP quantification as described in the literature include thermal degradation combined with gas chromatography–mass spectrometry (GC-MS) and additional methods that are extensively described elsewhere [67,68,69]. Different from these methods, the approach presented here can directly evaluate the mass concentration of the released plastics. The method is a simple, acceptably sensitive, and inexpensive option for analyzing the residues of small particulates, which are difficult to quantify on a particle number basis by µRaman and µFT-IR. However, a series of limitations can be found. To avoid signal saturation, the plastic particle size should be sufficiently small (i.e., below few microns) to let the IR beam pass through the sample. Moreover, the method is not as sensitive as the thermal degradation methods combined with GC-MS since for two samples we were not able to see any spectrum. In addition, the presence of more than one polymer or other contaminants can complicate the spectral evaluation and compromise the calibration curve linearity. All of these aspects need to be taken into account, eventually with a case-by-case approach, when exporting these methods to more complex samples.

Our findings were partially in agreement with what expressed by Busse at al. [70] in terms of mass of the nylon 6 released from teabags. However, the conclusions of the mentioned paper were that the material released from teabags was not consistent with true released particles (both pMP or pNP) but, rather, partially soluble oligomers.

We, therefore, hypothesized that what we observed during the µRaman investigation (Figure 1C) resulted from the precipitation of such oligomers due to the cooling process. Accordingly, we filtered the teabag leachates with different pore size membranes, either when hot immediately after boiling (“hot-filtered” leachate) or after cooling at room temperature (“cold-filtered” leachate). The results are summarized in Table 1.

Under “hot-filtered” conditions, it appears that the material present in the leachates passed through the filters independently from the cut off (except for the 0.2 µm filter). In particular, the mass of nylon 6 found after passing through the smallest pore size filter was not different from the overall quantity found when no filtration was applied (1.13 ± 0.07 mg of nylon 6, Cohen’s *d* 0.1). In contrast, if the leachate was cooled to room temperature before filtration, a different trend was noted. With a 2.0 µm cut off, almost all the material passed through the filter, while an increase in material retention was observed for smaller pore size filters. With a 0.02 µm cut off filter, approximately 65% of the overall material mass passed through the filter, meaning that about 35% of the total mass belonged to >20 nm particles. Table 1 reports the Cohen’s *d* values obtained by comparing the measured masses retrieved after filtration at the different cut-offs in the cold- or hot-filtered conditions. As can be seen, the highest difference in recovered mass was found at the smallest cut-offs (0.02 µm) that retained more materials in the cold-filtered conditions (Cohen’s d value 5.3, *p* value 0.003). On the contrary, with larger pore sized filters (0.4 µm and above) such variations were reduced, probably because the differences in filtered mass between the hot and cold conditions were not sufficient to determine a statistically relevant effect, as expected.

Analytical ultracentrifuge (AUC) investigations confirmed the presence of submicron particles suspended in the leachate after cooling (see Figure S10). These findings suggested that the nylon 6 released from the teabags during the heating process may actually be attributable to oligomers that are partially soluble when heated, as suggested by Busse et al. [70]. However, such oligomers, if allowed to cool before filtration, could precipitate from solution and aggregate or crystalize into submicron particles, which would be trapped during cold filtering. As an additional support for this finding, leachates after cooling were re-heated at 95 °C and immediately filtered with the smallest pore size filter (0.02 µm). The nylon 6 quantified in these samples was 1.01 ± 0.04 mg, meaning that the formed aggregates were able to dissolve again because of the heating process.

**Figure 2:**
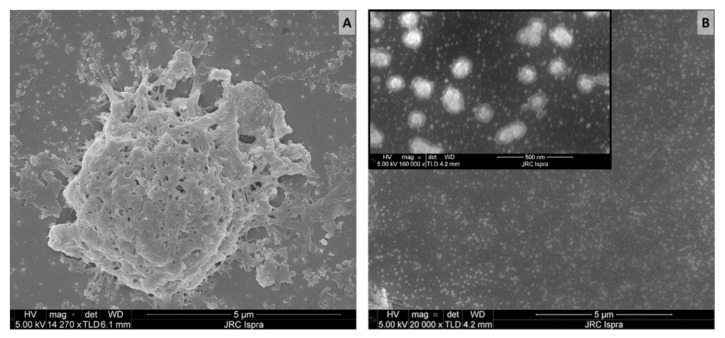
SEM images of teabag leachates prepared on different silicon substrates: (**A**) untreated Si (**B**) hydrophobically modified Si-PTFE-PDDA supports. Scale bar: 5 µm (500 nm for the insert in B).

An additional observation on the aggregation status of the released material and its importance in the quantification of pMP and pNP comes from the analysis of SEM images. SEM is one of the most used techniques for the investigation and counting of plastics, especially in the nano scale [48,69] but is critically dependent on appropriate sample preparation. If the sample preparation technique is not adequate, there is a high risk of obtaining misleading information. The first sample preparation approach was based on leachate spotted and dried directly on a polished silicon substrate that was previously cleaned with ethanol. From the resulting SEM image, a variety of particle sizes were detected (Figure 2A), including micron size particles and probable agglomerates, similar to what was already presented in the middle panel of Figure 1C.

To achieve a more homogeneous distribution on the surface, we took advantage of modified Si-PTFE-PDDA supports, which have been designed to have a surface with a controllably increased surface hydrophobicity. In these conditions, the particles bind to the surface by electrostatic forces, and their absorption is driven by diffusion. Unless particles aggregation occurs in the solution, no particle aggregation occurs on the surface [58]. As can be seen in Figure 2B, no microparticles were detected. This suggests that the micron-sized particles observed during the Raman investigation (Figure 1C) were actually an artifact of the sample preparation. In addition, the morphology of the micron-sized particles (Figure 2A) appears more similar to that of an aggregate than a single compact particle. If this is the case, particle counting on the basis of SEM images could introduce errors and huge variability with respect to the real number of particles present in the sample.

Together with the findings of the temperature effects on oligomer aggregation, it is clear that proper quantification or counting of pMP, and especially pNP, in diluted samples is still a challenge. The simple spotting of sample leachate on a silicon surface can help the visualization of particles and, eventually, their identification as demonstrated with the Raman investigation (Figure 1), but this is not sufficient to properly quantify the number of plastics released during sample preparation.

## 4. Conclusions

In conclusion, here we present a study on the release of pMP and pNP from different types of food packaging, i.e., PE rice of cooking bags and ice cube bags and nylon teabags. We demonstrated that the real use of the selected plastic packaging in everyday life can produce pMP and pNP detachment and consequent contamination of food for human consumption. The adaptation of multi-instrumental approaches already described in the environmental field was found to be fundamental for a complete characterization and a global understanding of what happens during the process. In particular, sample preparation was found to be a key point for the study of individual particles and to minimize the aggregation process. The introduction of the heating and cooling steps during the investigation revealed how this seems to affect the agglomeration status of the released oligomers, possibly inducing the formation of thermally unstable pNP. This finding poses challenging issues for legislators: should we consider keeping the heat-released material after cooling as being “oligomeric” or should we take into account the possible formation of pNP? The different ways in which these materials can be classified may potentially have different consequences when subjected to evaluation under different types of legislative controls.

## Figures and Tables

**Table 1:** Quantities of nylon 6 (mg) from teabag leachates after filtration with different pore sizes. The mass (mg) of nylon 6 quantified with the FT-IR method using KBr pellets on teabag leachates after filtration with different pore size filters immediately after teabag removal (“hot-filtered” leachate) or after cooling at room temperature (“cold-filtered” leachate). The values refer to the amount of nylon 6 found in the liquid portion recovered in the receiving flask (n = 3, data are presented as means ± standard deviations). In the last columns, the Cohen’s *d* and the *p* values for the two-sample *t*-test are reported.

Filter Pore Size µm	mg of Nylon 6 in Hot-Filtered Leachate	mg of Nylon 6 in Cold-Filtered Leachate	Cohen’s *d*	*p* Value (Two Sample *t*-Test)
0.02	1.13 ± 0.05	0.71 ± 0.10	5.3	0.003
0.2	0.60 ± 0.10	0.84 ± 0.14	1.9	0.080
0.4	0.91 ± 0.14	0.93 ± 0.03	0.2	NA ^1^
2.0	0.92 ± 0.08	1.03 ± 0.10	1.2	0.271

^1^ NA = not appropriate because Cohen’s *d* < 0.8, see description in the Materials and Methods Section.

## Data Availability

Not applicable.

## Supplementary Materials

The following supporting information can be downloaded at: https://www.mdpi.com/article/10.3390/polym14061238/s1, Figure S1: General scheme of the overall procedure for applying Beers law to Fourier transform infrared (FT-IR) spectroscopy; Figure S2: General scheme of sample preparation for suspensions after filtration with different pore sizes immediately after teabag removal or after cooling completely at room temperature; Figure S3: Chemical recognition by Raman microscopy on particles after filtration with Si_3_N_4_ filters; Figure S4: XPS spectra from the surface of the teabag net wire (reference) and the dropcast sample form the filtered nano-fraction from the teabag leachate; Figure S5: Elemental quantitative XPS analysis from the leachate nano-fraction sample and the teabag and substrate reference; Figure S6: XPS atomic ratios (O/C and C/N) for the leachate nanofraction and the teabag reference; Figure S7: XPS high-resolution spectra of the C1s peaks for the leachate nanofraction and the teabag reference; Figure S8: Nylon 6 spectra for calibration curve building; Figure S9: PE spectra for calibration curve building; Figure S10: Multimodal size distribution of the teabag leachate, as obtained from an analytical ultracentrifuge investigation; Table S1: Elemental quantitative XPS analysis from the leachate nano-fraction sample, and the teabag and substrate reference. Table S2: Quantities of nylon 6 (mg) realized from each single teabag after boiling. References [71,72,73] are cited in the supplementary materials.
