# Supplementary material for: Detecting Micro- and Nanoplastics Released from Food Packaging: Challenges and Analytical Strategies
Source: Polymers (Basel). 2022 Mar 18;14(6):1238. doi: 10.3390/polym14061238 (PMC8954753; doi:10.3390/polym14061238)
Supplement: Supplementary file 1 [file polymers-14-01238-s001.zip › polymers-1617879-supplementary.pdf]

polymers

MDPI

Supplementary Material

Detecting Micro- and Nanoplastics Released from Food Packaging: Challenges and Analytical Strategies

Claudia Cella, Rita La Spina, Dora Mehn, Francesco Fumagalli, Giacomo Ceccone, Andrea Valsesia and Douglas Gilliland *

European Commission, Joint Research Centre (JRC), 21027 Ispra, Italy; claudia.cella@ec.europa.eu (C.C.); rita.la-spina@ec.europa.eu (R.L.S.); dora.mehn@ec.europa.eu (D.M.); francesco-sirio.fumagalli@ec.europa.eu (F.F.); giacomo.ceccone@ec.europa.eu (G.C.); andrea.valsesia@ec.europa.eu (A.V.)

* Correspondence: douglas.gilliland@ec.europa.eu

Supporting methods

Detailed description of Fourier-Transformation InfraRed (FT-IR) spectroscopy applied to Beers law

In order to quantify the plastic material released from the food contact containers during use, we adopted a general approach of using Beer's law and FT-IR spectral analysis as follows. Each sample of residues was mixed with KBr (Sigma Aldrich) and pressed into a pellet which was analysed to produce an FT-IR spectrum to identify the polymer. Then, calibration curves for this polymer were prepared from FT-IR spectra obtained from pressed KBr pellets containing known amounts of finely ground pristine plastic. A calibration curve of the polymer was then generated from the integrated areas of specific peaks in the group frequencies region of the spectra. Finally, the integrated peak area in the spectrum of the residue was compared with the calibration and the resultant mass determined. The overall procedure is summarised in the following scheme (Figure S1):

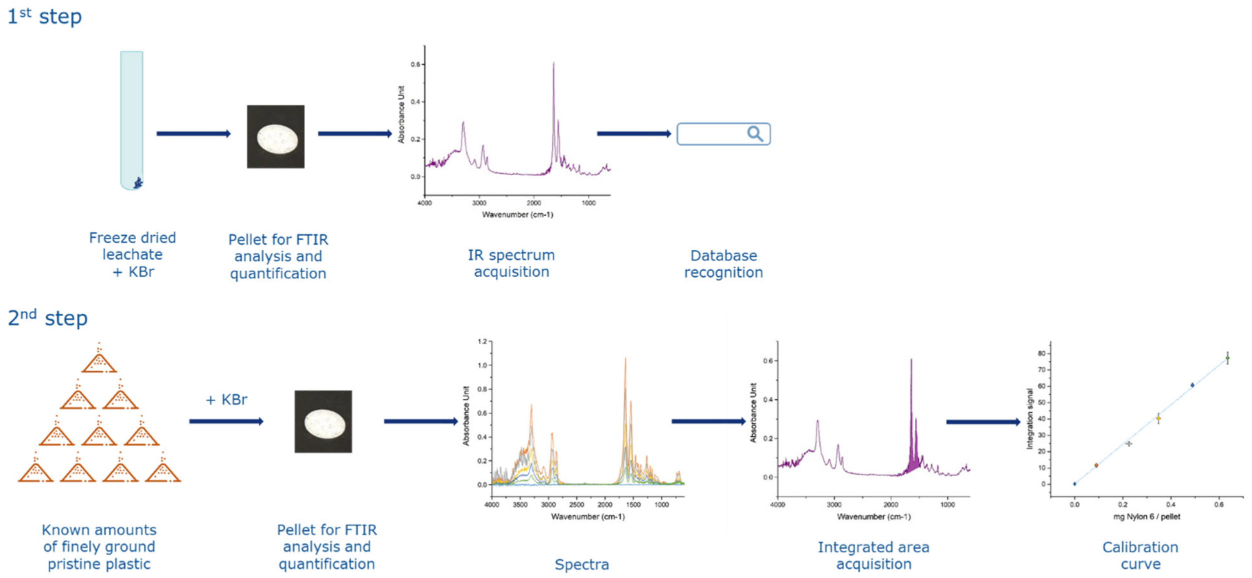

Figure S1. General scheme of the overall procedure for applying Beers law at Fourier-Transformation InfraRed (FT-IR) spectroscopy.

Sample preparation for suspensions after filtration with different pore size

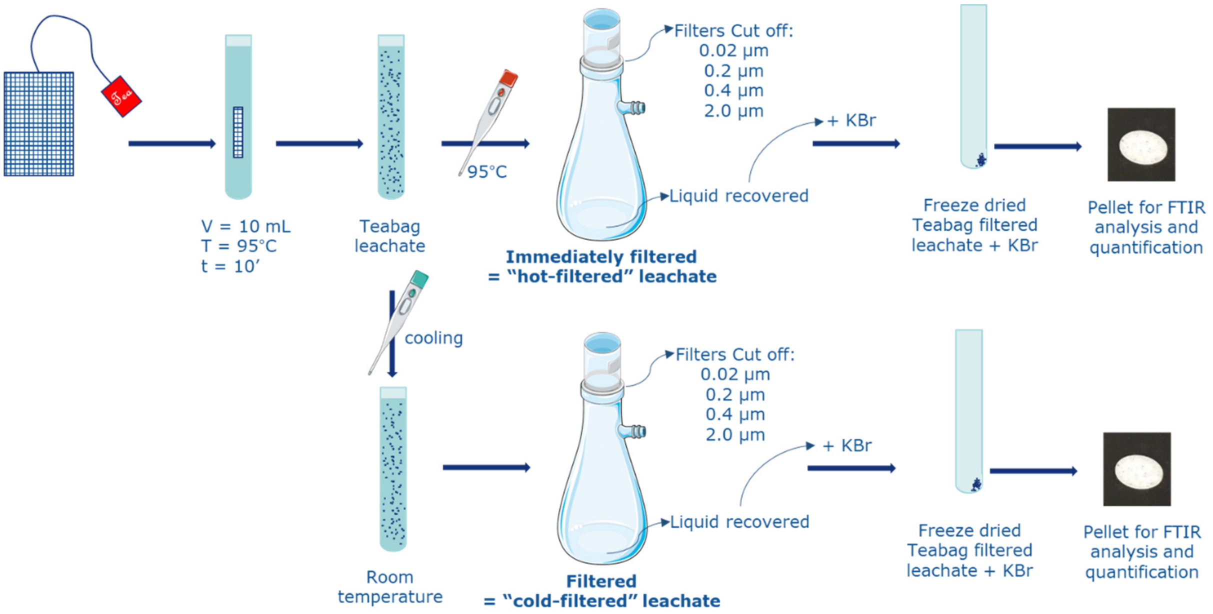

Figure S2. General scheme of sample preparation for suspensions after filtration with different pore size immediately after teabags removing or after complete cooling at room temperature.

Supporting Results

Chemical recognition by Raman microscopy on particles after filtration with Si_3N_4 filters

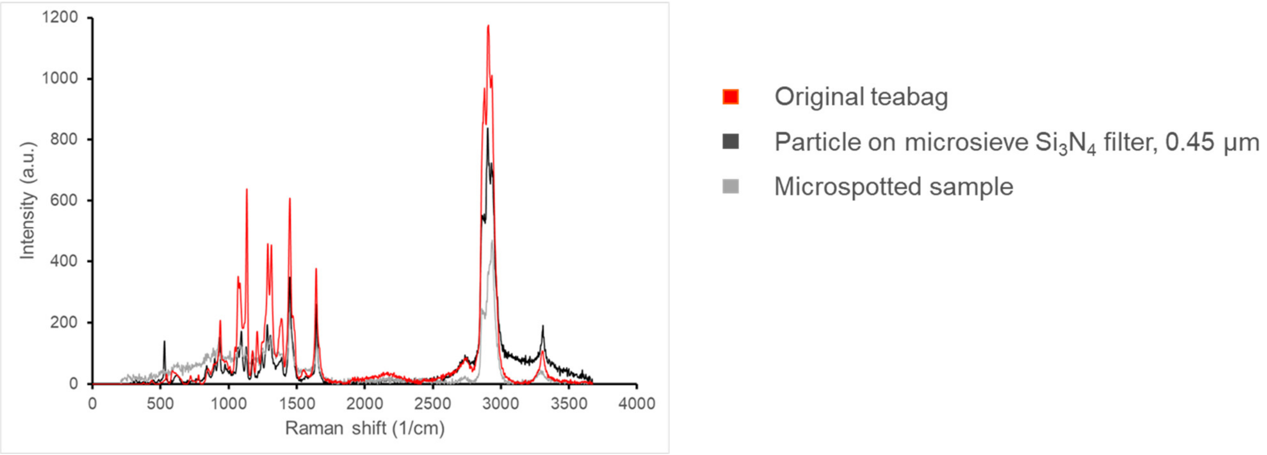

Figure S3. Chemical recognition by Raman microscopy on particles after filtration with Si_3N_4 filters (Aquamarijn, The Netherlands, pore size 0.45 µm). Particles on the filter resulted with black spectra, while particles leachate microspotted on silicon support gave the grey spectrum in the figure.

Detailed results for X-ray photoelectron spectroscopy (XPS)

Large spot sizes instead of micro-spotted ones allowed good S/N ratios to be obtain in the recorded spectra. Teflon strips as substrates minimize the uncertainties in the stoichiometric evaluation of the C content of the surface resulting from adventitious hydrocarbon contamination. The high binding energy shift of electrons originating from C-F bonds and the precisely known Teflon stoichiometry allows the substrate and sample contributions during the C 1s peak fitting to be separated with high confidence. Accordingly,

Teflon substrate contribution to the C 1s signal was not considered in the elemental quantification of the sample.

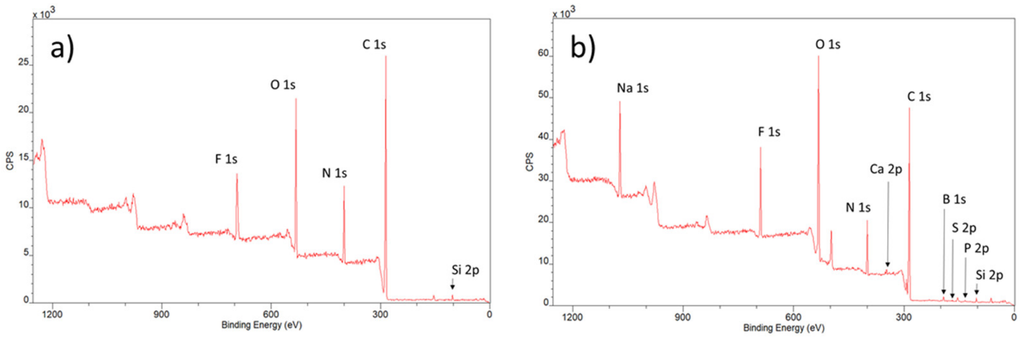

Figure S4. XPS spectra from a) the surface of the teabag net wire (reference) and b) dropcasted sample form the filtered a nano-fraction from the teabag leachate.

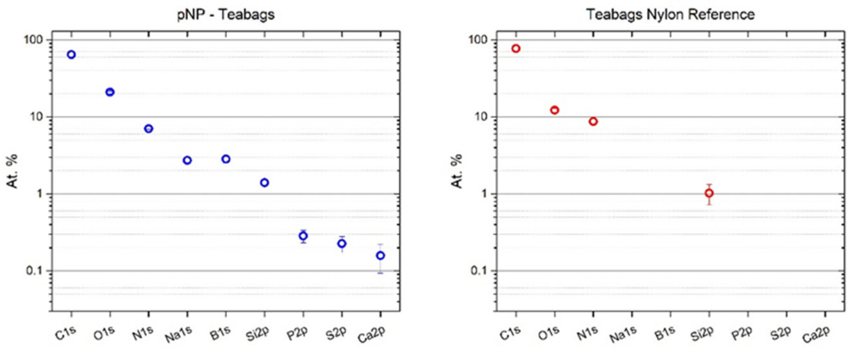

Figure S5. Elemental quantitative XPS analysis from the leachate nano-fraction sample, and the teabag and substrate reference.

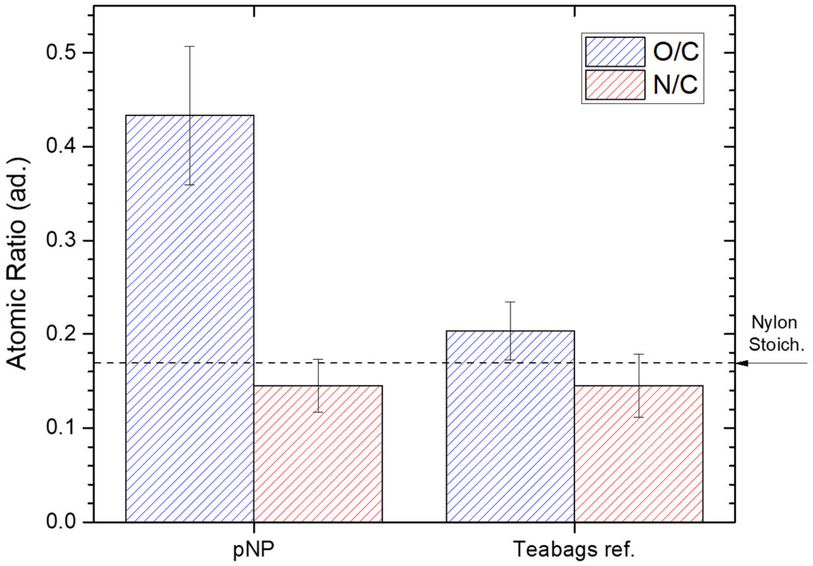

Figure S6. XPS atomic ratios (O/C and C/N) for the leachate nanofraction and the teabag reference.

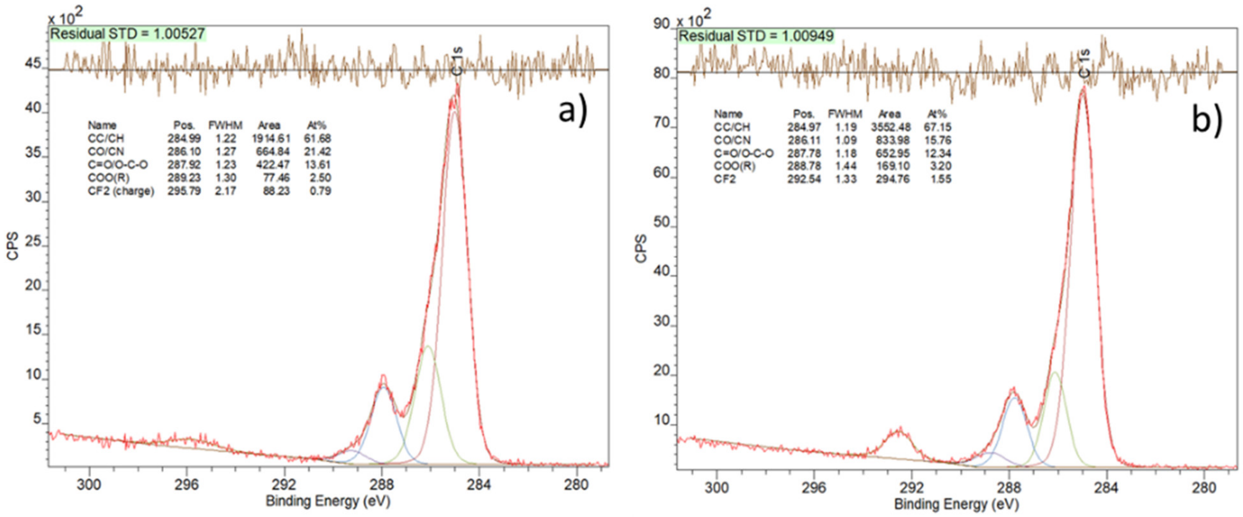

Figure S7. XPS high-resolution spectra of the C1s peaks for the leachate nanofraction and the teabag reference.

Nylon-6 FT-IR spectra and calibration curves

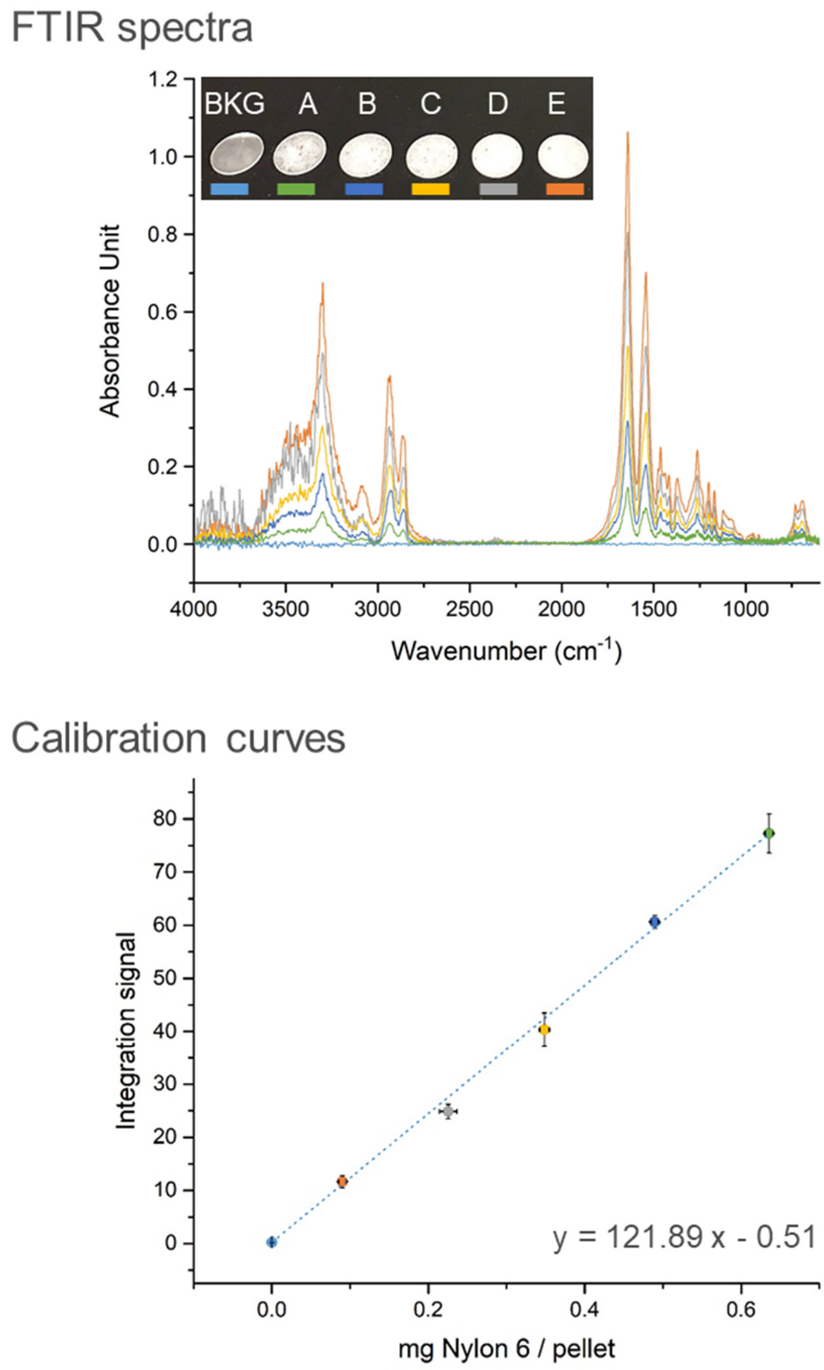

Figure S8. Nylon-6 spectra (top) for calibration curve building (bottom). In the insert, a picture of the KBr pellet with increasing mass of plastic. Bkg: 0.00 mg; A: 0.09 ± 0.00 mg; B: 0.23 ± 0.01 mg; C: 0.35 ± 0.01 mg; D: 0.49 ± 0.00 mg; E: 0.64 ± 0.01 mg. Standard deviations for the fitting coefficients intercept and slope (m,q) in the calibration curve were $\sigma_m = 0.04$ and $\sigma_q = 2.39$. Pearson's *r* was 0.99 and adjusted X^2 was 0.99.

PE spectra for calibration curve

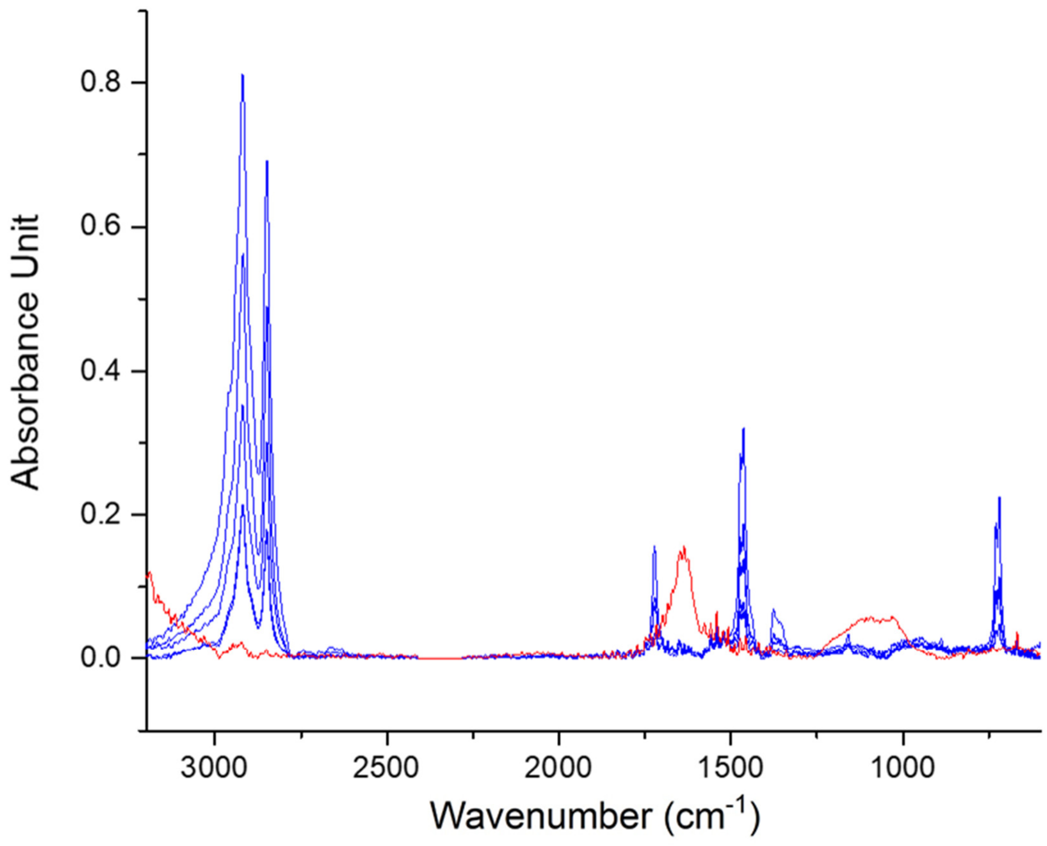

Figure S9. Calibration series of 2–0.13 mg/pellet polyethylene in KBr pellets (blue) and spectrum of the lyophilised fraction of the cooking liquid of rice bag (red). Signal of sample is below the detection limit.

Analytical ultracentrifuge (AUC) methods and results

AUC analysis was performed to measure the size distribution of teabag leachates in water using a Beckman Coulter ProteomeLabTM XL-I analytical ultracentrifuge with an 8 hole rotor, collecting optical interference signals. Measurement was run at 5000 rpm rotation speed at 20 °C. Data were fit using the ls-g*(s) model in the 1–200 S range, with a logarithmic spaced grid, resolution of 200 [71]. The bulk density of nylon 6 (1.14 g/mL) was applied when calculating size distribution from sedimentation coefficient distribution.

The release of particles in the nano range from the teabags leachate was additionally confirmed by AUC measurement. AUC has already been tested as a tool that allows to calculate particle size distribution from the sedimentation speed of the particles [72,73]. In the case of teabag leachate, AUC experiment confirmed the presence of sub micron sized particles. The multimodal size distribution of the sample with main peaks in the range 250–550 nm (radius) is shown in the figure below.

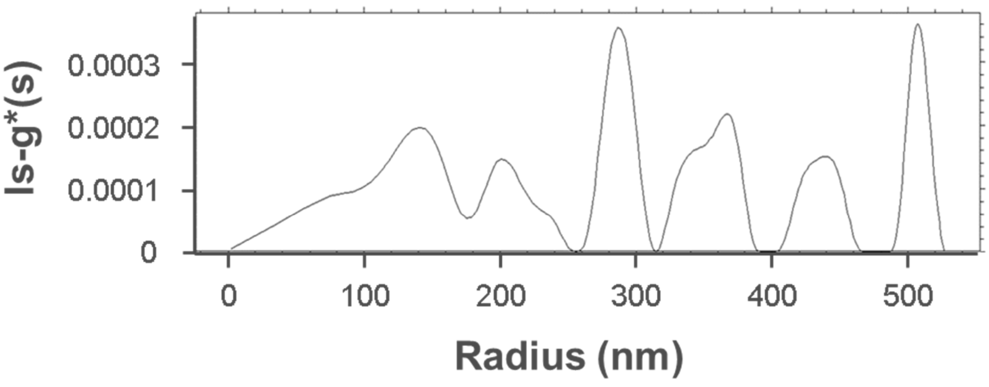

Figure S10. Multimodal size distribution of the teabag leachate, as obtained from analytical ultracentrifuge investigation.

71. Schuck, P .; Perugini, M.A.; Gonzales, N.R.; Hewlett, G.J.; Schubert, D. Size-distribution analysis of proteins by analytical ultracentrifugation: Strategies and application to model systems. *Biophys. J.* **2002**, 82, 1096–1111, doi:10.1016/S0006-3495(02)75469-6.
72. Mehn, D.; Capomaccio, R.; Gioria, S.; Gilliland, D.; Calzolai, L. Analytical ultracentrifugation for measuring drug distribution of doxorubicin loaded liposomes in human serum. *J. Nanoparticle Res.* **2020**, 22, doi:10.1007/s11051-020-04843-5.
73. Mehn, D.; Iavicoli, P.; Cabaleiro, N.; Borgos, S.E.; Caputo, F.; Geiss, O.; Calzolai, L.; Rossi, F.; Gilliland, D. Analytical ultracentrifugation for analysis of doxorubicin loaded liposomes. *Int. J. Pharm.* **2017**, 523, 320–326, doi:10.1016/j.ijpharm.2017.03.046.

Detailed results for X-ray photoelectron spectroscopy (XPS)

Table S1. Elemental quantitative XPS analysis from the leachate nano-fraction sample, and the teabag and substrate reference.

At%.	C1s	O1s	N1s	Na1s	B1s	Si2p	P2p	S2p	Ca2p	F1s
pNP – Teabag	64.38(5.94)	20.94(0.72)	7.02(0.12)	2.73(0.27)	2.84(0.29)	1.40(0.11)	0.28(0.05)	0.22(0.05)	0.16(0.06)	--
Teabag Ref.	77.41(7.66)	12.25(0.63)	8.71(0.76)	--	--	1.02(0.30)	--	--	--	--
Teflon	66.46(1.03)	--	--	--	--	--	--	--	--	33.54(1.03)

Quantities of Nylon-6 (mg) realised from each single teabags after boiling.

Quantities of Nylon-6 released (mg Nylon-6) were calculated by considering the actual mass of the pellet, the total mass of KBr added to the aliquot before freeze drying, the lyophilised aliquot volume and the total volume of liquid applied during the treatment of teabags.

Table S2. Quantities of Nylon-6 (mg) realised from each single teabags after boiling. .

	Integrated Area	Calculated Nylon-6 mg	Dilution Factor	mg Nylon-6
Background	0.24	-	-	-
Procedural blank	1.14	0.01	2.28	0.03
Sample 1	22.48	0.19	6.06	1.14
Sample 2	23.05	0.19	6.11	1.18
Sample 3	21.22	0.18	6.05	1.08
Sample 4	24.86	0.21	4.94	1.03
Sample 5	30.27	0.25	4.86	1.23
Sample 6	27.25	0.23	4.97	1.13
